# Mixture Design and Doehlert Matrix for Optimization of Energized Dispersive Guided Extraction (EDGE) of Theobromine and Caffeine from Cocoa Bean Shells

**DOI:** 10.3390/foods14050740

**Published:** 2025-02-21

**Authors:** Luciana Lordêlo Nascimento, Paulo Natan Alves dos Santos, Honnara Santos Granja, Larissa da Silveira Ferreira, João Victor Ferreira Lima, Bruna Louise de Moura Pita, Allan dos Santos Polidoro, Lisiane dos Santos Freitas, Elina Bastos Caramão, Fabio de Souza Dias, Alini Tinoco Fricks

**Affiliations:** 1 Programa de Pós-Graduação em Ciência de Alimentos, Universidade Federal da Bahia, Salvador 40170-115, BA, Brazil; lucianalordelo@outlook.com (L.L.N.); lsferreira03@gmail.com (L.d.S.F.); jvflima.agr@gmail.com (J.V.F.L.); 2 Rede Nordeste de Biotecnologia, Universidade Federal de Sergipe, São Cristóvão 49107-230, SE, Brazil; natan-9@hotmail.com (P.N.A.d.S.); lisiane_santos_freitas@yahoo.com.br (L.d.S.F.); elina@ufrgs.br (E.B.C.); 3 Programa de Pós-Graduação em Química, Universidade Federal de Sergipe, São Cristóvão 49107-230, SE, Brazil; ghonnara@gmail.com; 4 Departamento de Análises Bromatológicas, Faculdade de Farmácia, Universidade Federal da Bahia, Salvador 40170-115, BA, Brazil; brunalmpita@gmail.com; 5 Department of Chemistry, Pharmaceutical, and Agricultural Sciences, Università degli Studi di Ferrara, 44121 Ferrara, Italy; allan.spolidoro@gmail.com; 6 Instituto Nacional de Ciência e Tecnologia, Energia e Ambiente (INCT E&A), Salvador 40170-110, BA, Brazil; 7 Programa de Pós-Graduação em Química, Universidade Federal da Bahia, Salvador 40170-115, BA, Brazil; fsdias@ufba.br

**Keywords:** energized dispersive guided extraction (EDGE), cocoa waste valorization, methylxanthines, optimization, Doehlert matrix, green chemistry

## Abstract

This work describes the development of a method for the extraction of methylxanthines from cocoa bean shell (CBS) by employing the novel Energized Dispersive Guided Extraction (EDGE) system. The mixtures were composed of ethanol–methanol–water and the ratio was optimized using a simplex-centroid design. Doehlert design (DD) was used to optimize the variables of temperature and time while using methylxanthine content obtained by HPLC-DAD as an analytical response. The optimized mixture consisted of water–ethanol in a 3:2 ratio. The optimum operating conditions for extraction were achieved at a temperature of 148.5 °C and 382 s. Under optimal conditions, 20.14 mg g^−1^ DM of theobromine and 3.53 mg g^−1^ DM of caffeine were found in the CBS extract. Methylxanthines were quantified with good linearity, LOQs, LODs, precision, and accuracy. The EDGE system, a newly automated extraction instrument, has proven to be very efficient for the recovery of theobromine and caffeine, and is considered a green extraction procedure, as demonstrated by the analytical greenness metric for sample preparation.

## 1. Introduction

Approximately 5.020 tons of cocoa (*Theobroma cacao*) were grinded worldwide in 2022 [1] and the global cocoa market is projected to grow significantly in the coming years [2]. Cocoa grinding is a multistage process that involves the roasting, grinding, and refining of cocoa beans. The first step in this process involves roasting the beans. Once the beans are roasted, they are ground into cocoa nibs for further refinement into cocoa liquor. Moreover, cocoa bean shell (CBS), the external cover of cocoa beans, is the main by-product generated during this process, comprising approximately 10% of the total bean weight [3].

CBS is a rich source of methylxanthines such as theobromine (3,7-dimethylxanthine) and caffeine (1,3,7-trimethylxanthine) [4,5]. Theobromine is recognized as a mild central nervous system stimulant, lacking the dependency risks often associated with caffeine. It has also been investigated for its anti-inflammatory effects, potential to mitigate neurodegenerative disorders, and neuroprotective properties. Caffeine, as the most widely consumed psychoactive substance globally, holds significant economic value [6,7].

Methylxanthines are typically extracted from plant materials using a variety of conventional and modern extraction methods. Conventional methods, such as percolation, maceration, hydrodistillation, and Soxhlet, require large amounts of solvents, are labor-intensive, and require considerable time. In contrast, modern methods like supercritical fluid extraction (SFE), microwave-assisted extraction (MAE), ultrasound-assisted extraction (UAE), and pressurized liquid extraction (PLE) overcome these challenges [8,9].

Recently, the Energized Dispersive Guided Extraction (EDGE) system, developed by combining dispersive solid-phase extraction with pressurized liquid extraction, has emerged as an alternative to Accelerated Solvent Extraction. It is considered faster than Soxhlet, more automated than Quick, Easy, Cheap, Effective, Rugged and Safe extraction (QuEChERS), and simpler than other solvent extraction systems, with eco-friendly attributes like reduced solvent use and shorter operation times [10,11]. Moreover, the assessment of the greenness of an extraction should be performed in accordance with the ten principles of sample preparation [12], which can be measured using metric tools such as AGREEprep [13], an open-access analytical greenness metric for sample separation.

In this context, several authors report the optimization extraction of bioactive compounds employing this technique. Tasfiyati et al. [14] optimized the extraction of scopoletin from *Morinda citrifolia* L. fruits using a three-factor central composite design (CCD) with the EDGE system. Souza et al. [15] applied a two-factor CCD to enhance the extraction of hinokinin from the dried bark of *Commiphora leptophloeos* Mart.-J. B. Gillett, using EDGE and GC-MS for analysis. Similarly, Dos Santos et al. [8] optimized the extraction of antioxidants from *E. uniflora* leaves using EDGE and response surface methodology.

Moreover, the selective extraction of bioactive compounds depends on several operating parameters, and designing the experimental runs is tedious and time-consuming if performed manually. Thus, the Design of Experiments using Response Surface Methodology (DoE-RSM) is a technique that not only plans the experiments with the least possible number of experimental runs but also gives a correlation between input and output variables [16]. DoE optimizes two types of variables: process variables, with matrices like Box–Behnken (BB), Doehlert design (DD), and Central Composite Designs, and mixture variables, which must sum to 1 or 100% and generate responses based on component proportions [17].

Among these, the DD has stood out for being quite economical, versatile, and efficient in modeling experimental data, in addition to offering very interesting flexibility in the choice of levels of the variables under study. Compared to CCD and BB, the DD stands out for requiring fewer experimental points, making it more efficient [18]. On the other hand, the mixture designs (MDs), such as simplex-centroid, allow the investigation of synergistic or antagonistic effects of the mixture components on the response variables [19].

Furthermore, neither DD nor MD has been applied to optimize the EDGE system, and the greenness of this technique remains unaddressed. Based on this assumption, this study aimed to develop an optimized methodology to obtain CBS extracts with high theobromine and caffeine content using the EDGE system. The process involved (1) employing a simplex-centroid MD to optimize the extractive solution for maximum methylxanthine content, (2) using a DD for parametric optimization, and (3) assessing the greenness of the extraction method with the AGREEprep metric tool.

## 2. Materials and Methods

### 2.1. Plant Material and Chemicals

CBS was donated by an organic chocolate factory located in Salvador, Bahia, Brazil, according to the following geographic coordinates: latitude −12°54′13.6728″, −38°26′34.3926″. The CBS was milled and sieved through a 20 mesh (0.841 mm). In addition, the CBS was defatted using hexane. Chromatography-grade ethanol and hexane were obtained from J. T. Baker (Phillipsburg, NJ, USA). The standard 2,2-Diphenyl-1-picrylhydrazyl (DPPH), 2,2′-azinobis (3-ethylbenzothiazoline-6-sulfonic acid) (ABTS), Trolox, gallic acid, caffeine, and theobromine were purchased from Sigma Aldrich, as well as Folin–Ciocalteu reagent, potassium persulfate, and sodium carbonate.

### 2.2. EDGEs

CBS (500 mg) was directly weighed into a Q-Cup containing an S1 Q-Disc filter set (C9 + G1 + C9), where C9 was a cellulose filter (pore size of 40 µm) and G1 was a fiberglass filter (pore size of 0.3 µm). Q-cups and collection vials were placed in an EDGE removable rack. Extracts were obtained using 20 mL of 40% (*v*/*v*) ethanol. After each extraction, a washing step (10 mL, 180 °C, 30 s) was performed with the same solvent to clean the system before the extraction of the next sample. The extracts were collected and stored at −8 °C until further analysis.

### 2.3. Optimization Strategy

**Figure 1:**
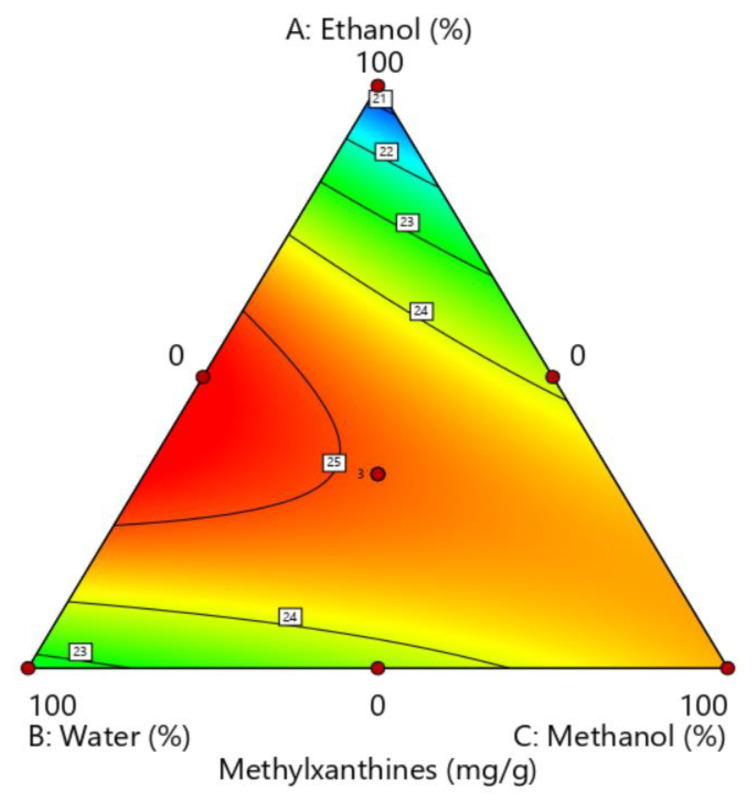
Contour lines of the analytical signal as a function of the mixture ingredient proportions for this model.

The optimization process was performed in two steps. First, a simplex-centroid design with pure ethanol, methanol, and water was applied to optimize the proportion of solvent for the extraction of the maximum methylxanthine response in the EDGE system. The methylxanthine content was given by the sum of theobromine (mg g^−1^ DM) and caffeine (mg g^−1^ DM) obtained by HPLC. The compositions used in the mixture design and the analytical signals are listed in Table 1, and the generated graph is shown in Figure 1. The second step in the optimization process was to determine the optimal conditions for the independent variables of temperature (°C) and time (s) in the EDGE system by applying Doehlert design, while using the methylxanthine content obtained by HPLC as the analytical response. Table 2 shows the factors, levels, and experimental matrix of the Doehlert matrix with the respective response for each run. All experimental data were processed using Design Expert 13 [20].

### 2.4. Liquid Chromatographic Analysis

Methylxanthines present in the extracts obtained using the EDGE system (Table 1 and Table 2) were identified and quantified by liquid chromatography. Samples were analyzed using an HPLC model LC20AD (Shimadzu, Tokyo, Japan) system consisting of an autosampler and diode array detector (DAD). An RP-C18 column, 4.6 × 150 mm with porosity of 5.0 μm (Phenomenex, Torrance, CA, USA), and a guard column (4.6 mm ID × 12.5 mm) were employed. The oven temperature was maintained at 45 °C. The mobile phase was composed of water–acetic acid (99:1) (Solvent A) and acetonitrile (Solvent B) at a flow rate of 1.0 mL min^−1^. An isocratic mode was employed using 80% solvent A. UV absorbance was monitored from 200 to 400 nm. Identification was performed by comparing the retention times of CBS extracts with those of the corresponding standards. Methylxanthines (mg/g DM) were quantified at 273 nm based on the integrated peak areas of the samples and standards (Sigma-Aldrich, Berllin, Germany) using external calibration.

### 2.5. Validation Procedures

The proposed HPLC method was validated in terms of linearity, limit of detection (LOD), limit of quantification (LOQ), precision, and accuracy. The linearity of the method was established through the external standard technique, and a calibration curve was developed using eight different concentrations (1–100.0 mg L^−1^) of standard solutions of theobromine and caffeine, which were measured seven times, and the peak area was plotted against the concentration. The LOD was calculated according to the following equation: 3.3σ/S—where σ is the standard deviation of the y-intercept values and S is the mean slope value from the constructed calibration curves. The LOQ value is 10σ/S [21].

Evaluation of precision was conducted in terms of repeatability (intraday precision) and intermediate precision (interday precision). Precision was expressed in terms of relative standard deviation (RSD). Repeatability was estimated by injecting standard solutions of theobromine and caffeine seven times on the same day at two concentrations (10 mg L^−1^ and 30 mg L^−1^). Intermediate precision was determined by injecting the same solutions employed in the repeatability test seven times across two days. The accuracy was determined by spiking the extract obtained under optimal conditions with standard samples of theobromine and caffeine at two concentration levels (10 and 30 mg L^−1^). Accuracy was expressed as a recovery percentage in relation to each analyte.

### 2.6. Analytical Greenness Metric Tool

The AGREEprep open-access software [13] was used to assess the greenness of the proposed hydroalcoholic extraction from CBS using the EDGE system. This metric tool has been recently introduced to study the environmental impact of the sample preparation stage and is based on ten consecutive steps of assessment that correspond to the ten principles of green sample preparation. Each step is evaluated and scored and the result is a pictogram representing the assessment of each step, together with an overall score on a scale ranging from 0 (considered a non-environmentally friendly approach) to 1 (the ideal greenest approach). In addition, each step is assigned an importance or weight to the total score and can be modified as desired. In this study, the weights of each step were set to their default values and the input data were compiled according to Pena-Pereira et al. [22].

### 2.7. Determination of Total Phenolic Content (TPC)

The TPC of the extracts was determined using the Folin and Ciocalteu method [23], with some modifications. A UV-Vis spectrophotometer (FEMTO 800 XI, São Paulo, Brazil) was used to obtain samples and standard readings at 760 nm. The extract (20 µL) was mixed with 500 µL of the Folin and Ciocalteu phenolic reagents. After 3 min, 5 mL of sodium carbonate solution (7% *w*/*v* in distilled water) was added to the mixture. The reaction was performed in the dark for 2 h and the absorbance was measured at 760 nm. The phenolic content was calculated as milligrams of gallic acid equivalents per gram of dry plant material (mg GAE/g DM), based on a standard curve of gallic acid [24].

### 2.8. Determination of Antioxidant Activity

#### 2.8.1. DPPH Radical Scavenging Analysis

The DPPH radical scavenging capacity was determined according to the modified DPPH free radical scavenging method developed by Brand-Williams, Cuvelier, and Berset [25]. Thus, 0.1 mL of the adequately diluted sample was mixed with 2.9 mL ethanolic DPPH solution. The mixture was then incubated for 30 min at an ambient temperature in the dark. A UV-Vis spectrophotometer (FEMTO 800 XI, São Paulo, Brazil) was used to obtain the samples and standard readings at 515 nm. Trolox reagent was used as a standard, and the results are expressed as milligrams of Trolox equivalent per gram of dry plant material (mg TE g^−1^ DM^−1^).

#### 2.8.2. ABTS Radical Scavenging Analysis

The ABTS free radical decolorization assay was performed according to the method described by Mellinas et al. [26], with slight modifications. The radical monocation of ABTS was generated by the reaction of the ABTS solution (7 mM) with 2.45 mM potassium persulfate at room temperature for 16 h in the dark. The solution was then diluted with absolute ethanol until the absorbance of 0.70 ± 0.02 at 734 nm. The sample (150 μL) was mixed with ABTS solution (2850 μL). A UV-Vis spectrophotometer (FEMTO 800 XI, São Paulo, Brazil) was used to obtain the samples and standard readings at 593 nm. Trolox was used as the standard to construct a calibration curve. The results are expressed as milligrams of Trolox equivalent per gram of dry matter (mg TE g^−1^ DM).

## 3. Results

### 3.1. Optimization of the Extractor Solution

A simplex-centroid using pure solvents (ethanol, water, and methanol) was developed to optimize the extractor solution composition using the total methylxanthine content obtained by HPLC as a response (Table 1).

Least-squares regression was used to adjust the linear, quadratic, and special cubic components to the analytical signal as a function of the mixture component proportions. Analysis of variance (ANOVA) was performed to test for the lack of fit and significance of the fitted models. A quadratic model was used to obtain a better description of the experimental critical region. The adjusted model was significant (*p* = 0.0012 < 0.05) and showed no lack of fit (*p* = 0.2739 > 0.05). The coefficient of determination (R^2^) and adjusted coefficient of determination (R^2^ adj) for the model were 0.9949 and 0.9863, respectively. The quadratic model equation is given below:

Methylxanthine content = 0.2054 × (%ethanol) + 0.2269 × (%water) + 0.2449 × (%methanol) + 0.0015 × (%ethanol) (%water) + 0.00053 × (%ethanol) (%methanol) + 0.00003 × (%water) (%methanol)(1)

The concentration of methanol was the most significant term among the linear factors, followed by water and ethanol. All interactions were significant except for the interaction between water and methanol. Pure ethanol and pure water presented the lowest analytical signal, whereas their combination presented the highest signal, showing positive effects between ethanol and water interactions. The polarity of the solvent affects the extractability of methylxanthines from plant materials. Methylxanthines are more soluble in high-polarity solvents, such as water, than ethanol and methanol [27]. Viscosity also plays a key role in plant extraction, by affecting the kinetics and efficiency of the extraction process. Low-viscosity solvents such as methanol and ethanol increase the diffusion rate and mass transfer of solutes from the plant material to the solvent. Typically, the viscosity of an ethanol–water mixture is lower than that of pure water, especially at intermediate ethanol concentrations. Therefore, the high analytical signal obtained in this study for the extractor solution containing 50% ethanol and 50% water can be explained by the synergistic effects between ethanol and water.

Equation (1) indicates that the critical point is the saddle point of the response surface and that these coordinates should not be used to determine the optimal conditions. Therefore, to determine the best proportions for obtaining a higher level of extraction, a visual inspection of the contour chart (Figure 1) was necessary. The extractor solution consisted of a mixture of 60% water and 40% ethanol.

Hydroethanolic extraction is often used to extract methylxanthines. Santana and Macedo [28] studied the effects of hydroalcoholic extraction on the recovery of catechins and methylxanthines from guarana waste using univariate analysis. The highest extraction yield was obtained using a mixture of 50% water and 50% ethanol, with particle sizes of 125 µm and 1.68 mm. Machado et al. [29] proposed a univariate evaluation of four different solvent systems for the extraction of methylxanthines and phenolics from Brazilian guaraná (*Paullinia cupana* Kunth), as well as ethanol, methanol, a mixture of ethanol and water (8:2), and ethanol–ethyl acetate (8:2). For the electromagnetic stirred extraction of theobromine, no significant differences were observed between pure methanol and ethanol–water (8:2), whereas pure methanol was the best solvent for caffeine extraction. Barreto et al. [27] proposed a mixture design for the extraction of methylxanthines and catechins from guarana seeds using ultrasound assisted extraction and the optimal extractor solution was composed of 72.5% water, 22.5% ethanol, and 5.0% acetone.

### 3.2. Optimization of EDGE Conditions

In this study, a Doehlert matrix was used to optimize the experimental conditions for the extraction of methylxanthines using EDGE, whereas HPLC-DAD was used to quantify theobromine and caffeine (Table 2).

Models (linear and quadratic) were fitted to the obtained methylxanthine content, and analysis of variance (ANOVA) at a 95% confidence level was used to evaluate the adequacy of the fitted models. The quadratic model best described the data behavior; it was highly significant (*p* < 0.0001) and did not show a lack of fit (*p* = 0.0655 > 0.05). Thus, the obtained model fits well with the data collected by the Doehlert design and can be represented by the quadratic model shown in Equation (2):

Methylxanthine content = −222.412 + 2.36668 × A + 0.382496 × B + 0.000315718 × AB − 0.00839135 × A^2^ − 0.00056204 × B^2^ (2)

where methylxanthine content represents the sum of theobromine and caffeine, A is the temperature (°C), and B is time (s).

Outstanding values were obtained for the coefficient of determination (R^2^) and adjusted coefficient of determination (R^2^ adj) for the model (0.9999 and 0.9996, respectively), indicating good agreement between the experimental and predicted values. The obtained model was also evaluated by comparing the predicted (Vp) and experimental (Ve) values. There was a good correlation between these values, confirming that the model fits well with the experimental data obtained from the Doehlert design, according to Equation (3):

Vp = 0.9991 × Ve + 0.02324 (R^2^ = 0.9998) (3)

**Figure 2:**
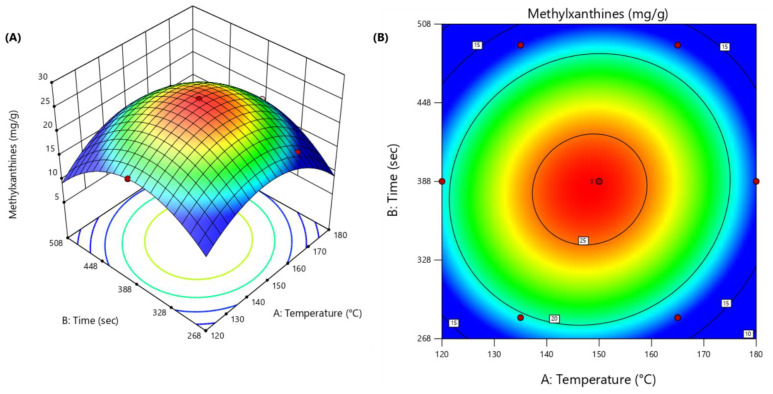
Response surface (**A**) and contour charts (**B**) obtained from Doehlert design.

The optimal value for each variable was determined via response surface methodology using a Doehlert design (Figure 2) [18]. The model showed a maximum response for the following experimental conditions (critical points): temperature = 148.5 °C, time = 382 s. Under these conditions, the model predicted 25.99 ± 0.077 mg/g of methylxanthines. The suitability of the proposed model for optimal prediction was evaluated by comparing its results with those obtained from a triplicate of experiments. The mean experimental value of methylxanthine content was 23.67 ± 0.051 (20.14 mg/g DM of theobromine and 3.53 mg/d DM of caffeine, respectively), presenting a difference of 8.9%. This result attests to the quality of model predictions.

### 3.3. Effect of EDGE Cycles on Methylxanthines, Phenolic Content, and Antioxidant Activity

Under optimal conditions, exhaustive extraction was performed by multiple extractions using the same sample in the EDGE system. Given the limited number of studies on this subject using EDGE, serial exhaustive extraction was found to be suitable for evaluating the methylxanthine content profile of CBS extracts over cycles. The theobromine content in the first EDGE cycle was 20.14 mg/g DM (optimal condition), while it was 1.78 mg/g DM in the second cycle and only 0.29 mg/g DM in the third cycle (Table 3). For caffeine, 3.53 mg/g DM (optimal condition) was found in the first cycle, while 0.26 mg/g DM was found in the second cycle and only 0.04 mg/g DM was found in the third cycle.

TPC was also assessed under cycles at optimal extraction conditions. TPC ranged from 80.10 to 1.88 mg GAE/g DM, in the first and third cycle, respectively. The TPC of the first cycle of extraction using EDGE was 42 times higher than that of the third cycle. In addition, the first cycle resulted in significantly higher antioxidant activity values than the other cycles, in accordance with the TPC values.

Two different methods were used to evaluate the antioxidant capacity of the extracts obtained under optimal conditions: ABTS and DPPH radical-scavenging methods. When compared to other studies, CBS extracts from the CCN-51 cocoa variety presented 171.32 mg TE g^−1^ DM (ABTS), whereas CBS from the Trinitario type of cocoa from Ecuador presented a value of 167.06 mg TE g^−1^ DM (ABTS) [30]. Much lower values were obtained by Benítez-Correa et al. [31] using conventional extraction with 70% ethanol, around 10 mg TE g^−1^ DM (DPPH). Antioxidant activity is influenced by multiple elements, including sample preparation, the extraction method used, the type of solvent, temperature, pH level, concentration of antioxidant compounds, the makeup of individual phytochemical components, and the chemical makeup of antioxidant molecules [32].

Overall, given the solvent type, temperature, and time conditions used in this study under optimal conditions, one extraction cycle using the EDGE system should be considered sufficient to recover most bioactive compounds present in CBS. The use of extraction cycles in the EDGE system was investigated by N. A. dos Santos et al. [8] during the extraction of bioactive compounds with antioxidant activity from Pitanga leaves, and the optimum operation conditions were achieved with one cycle of extraction.

### 3.4. Analytical Performance

The proposed HPLC method was validated in terms of linearity, limit of detection (LOD), limit of quantification (LOQ), precision, and accuracy (Table 4). The regression statistics were calculated from the calibration curves constructed for theobromine and caffeine and showed satisfactory linearity with a correlation coefficient (R^2^) > 0.99. The calculated F values of the regression analysis were 4713.7 for theobromine and 6109 for theobromine and caffeine, respectively, which were higher than the critical F value (F_1,3_ = 10.13) at a 95% confidence level, demonstrating that the regression was significant. This finding indicates that theobromine and caffeine can be determined using the proposed extraction method using external calibration standards. External calibration indicates the reliability and practicality of a method.

The limit of detection (LOD) and limit of quantification (LOQ) were determined using calibration curve data (Table 4). The repeatability (intraday precision) and intermediate precision (interday precision) of the method were expressed as relative standard deviations (RSDs) and were assessed based on the results of seven replicates of the two different concentrations of analytes. Table 4 presents the average results. Theobromine showed low intraday RSD values (10 mg L^−1^: 0.88%; 30 mg L^−1^: 0.23%) and low interday values (10 mg L^−1^: 0.32%; 30 mg L^−1^: 0.15%). Similarly, caffeine presented acceptable intraday (10 mg L^−1^: 0.82%; 30 mg L^−1^: 0.49%) and interday (10 mg L^−1^: 0.39%; 30 mg L^−1^: 0.95%) RSD values.

The average accuracy results are listed in Table 4. The recoveries were performed with the addition of the standard to the sample and the concentration was determined using the proposed method. The recovery percentage was calculated using Equation (4):

(%) = (Cs − Co)/Cadd(4)

where Cs is the result of the spiked samples, Co is the result of the unspiked samples, and Cadd is the amount of analyte added. The recoveries ranged between 103.0% and 106.7% for theobromine and between 84.9% and 118.9% for caffeine, respectively. Therefore, it can be stated that the proposed extraction procedure using the EDGE system is suitable for the extraction of polyphenolic compounds in food samples.

### 3.5. Comparison with Other Extraction Methods

**Figure 3:**
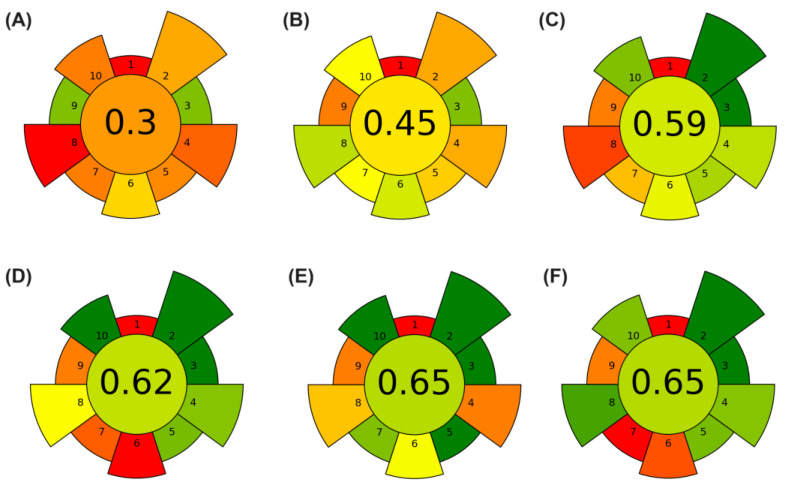
Green assessment results with AGREEprep of the extraction methods for the determination of methylxanthines in cocoa bean shell: (**A**) hydrothermal hydrolysis; (**B**) microwave-assisted extraction; (**C**) pressurized liquid extraction; (**D**) magnetically stirred extraction; (**E**) this work; (**F**) ultrasound-assisted extraction.

The greenness of the extraction proposed in this study was evaluated and compared with other previously reported non-conventional extraction methods [5,26,31,33,34] for analytes from CBS using the AGREEprep metric tool. As can be seen from the assessment results shown in Figure 3, the two extraction procedures had a final score below 0.5. Among them, the hydrothermal hydrolysis of CBS [34] seems to be the least green method, with a final score of 0.3 (Figure 3A), mainly because of the high volumes of hazardous solvents and reagents (criteria 2 and 10), the amount of waste generated (criterion 4), and energy-intensive instrumentation (criterion 8). Because of its low score, this extraction procedure was not considered green according to the ten principles of green chemistry [12,35]. The highest amount of theobromine (9.4 mg g^−1^ CBS DM) and caffeine (3.5 mg g^−1^ CBS DM) was obtained with samples treated at pH 4 during 13 min.

A score of 0.45 (Figure 3B) was assigned to a method based on microwave-assisted extraction [26], mainly because of the use of hazardous solvents (criteria 2 and 10) to adjust the pH and generate a large amount of waste (criterion 4). AGREEprep assigns scores of 1.0 to methods that produce less than 0.1 g (or milliliters) of waste and scores of 0.0 to methods that generate more than 50 g (or milliliters) of waste. For waste amounts that fall between these two levels, AGREEprep employs a logarithmic function to calculate scores [22]. Mellinas et al. [26] proposed a Box–Behnken design to evaluate the effects of pH, time, temperature, and solid–liquid ratio on the extraction yield, total uronic acid content, total phenolic content (TPC), and antioxidant performance of CBS using microwave-assisted extraction (MAE). Optimal MAE conditions were determined as 5 min, pH 12, 97 °C, and solid–liquid ratio of 0.04 g mL^−1^, and these showed a better outcome than conventional solvent extraction. Theobromine was the major analyte obtained under the optimal conditions (7.62 mg g^−1^).

A score of 0.59 (Figure 3C) was assigned to Pagliari et al. [36], who proposed a Behnken design to evaluate the effects of temperature (90–130 °C), number of cycles (1–5), modifier (EtOH 0–15%), and static time (2–6 min) on theobromine, caffeine, and antioxidant activity during pressurized hot water extraction. Under optimized conditions (ethanol 15%, temperature 90 °C, five cycles and static time 6 min), theobromine and caffeine content were, respectively, 20.6 ± 0.06 and 4.8 ± 0.02 mg g^−1^ DM. The extraction process achieved a “medium” green rating level. The main drawback of this process is its high energy consumption (Criterion 8).

A score of 0.62 (Figure 3D) was assigned to magnetically stirred extraction, using deep eutectic solvents [31], which is considered a medium-green extraction of bioactive compounds from CBS. The main limitation of this method from a green chemistry perspective is the excessively long extraction time (criterion 6) with a multitude of consecutive steps (criterion 7) and, consequently, higher energy consumption instrumentation (criterion 8). Benítez-Correa et al. [31] proposed a univariate approach to investigate classic solid–liquid extraction using non-conventional choline chloride-based deep eutectic solvents (ChCl-DES). The theobromine and caffeine concentrations ranged from 2.59 to 5.80 mg g^−1^ DM and 0.87–2.08 mg g^−1^ DM, respectively.

The greenest extraction methods were the EDGE system proposed in this study (Figure 3E) and the ultrasound-assisted extraction proposed by Ramos-Escudero et al. [33] (Figure 3F), both with an overall score of 0.65. Regarding EDGE, a good score was achieved mainly because of the lack of volume of hazardous solvents and reagents (criterion 2), use of sustainable and renewable materials (criterion 3), low amount of sample used (criterion 5), lack of physical hazards (criterion 10), automation of the system (criterion 7), and operator safety aspects (criterion 10). However, the amount of waste generated (criterion 4) and energy-intensive instrumentation (criterion 8) are the main limitations of the proposed method. After extraction, the EDGE software system was programmed to clean with 10 mL of ethanol, thus generating waste after extraction. In addition, the EDGE equipment has a maximum power of 1000 W, which was considered for the energy consumption calculation. Compared to sonication [33], the EDGE system presents a much higher energy demand. One of the main drawbacks of the ultrasound method proposed by Ramos-Escudero et al. [33] was the long extraction time (criterion 6), especially considering ultrasound pulse intermittent, with a multitude of consecutive steps (criterion 7). In their study, a Box–Behnken design was proposed to evaluate the effects of time, amplitude, and ethanol content on the ultrasound-assisted extraction of bioactive compounds from CBS. Under optimal conditions (15 min of sonication, 80% amplitude, and 50% (*v*/*v*) ethanol), 39 compounds were identified by LC-ESI-qTOF-MS. Theobromine was identified but not quantified, whereas caffeine was not identified in the sample. From a green perspective, ultrasound-assisted extraction has been reported to be ecologically friendly when considering the factors of energy consumption and CO_2_ emissions, in addition to being considered a low-cost technology [35].

A common aspect of all the methods assessed here is that they do not meet criterion 1, which favors in situ sample preparation. However, in general, this is hardly fulfilled [37]. In addition, almost all the methods assessed here had a negative impact on their score based on criterion 9, which relates to the subsequent instrumentation used (criterion 9). A higher score is given to readily available detection systems, whereas a lower score is given to the use of liquid or gas chromatography because of the higher demand in terms of energy consumption and reagents, which shifts the environmental impact from the sample preparation to the determination step.

The assessment of greenness using the AGREEprep tool allows the evaluation of the environmental impact of the sample preparation step. Thus, according to the results obtained in this study, the proposed sample preparation method using the EDGE system can be considered aligned with the principles of green sample preparation [12]. In terms of analytical performance, the quantitative results for caffeine and theobromine were consistent with the range of data reported in the literature for cocoa shells. In particular, the results obtained in this study were very similar to those obtained by Pagliari et al. [5], but much higher than those of other studies [26,31,38]. Grillo et al. [4] obtained a higher theobromine content using hydrodynamic cavitation (HC), but lower caffeine content. They used an HC reactor for 3.93 min with a ternary water/ethanol/hexane mixture, providing a hydrophilic product rich in methylxanthines and polyphenols, and a lipid layer. They obtained theobromine content of 32.7 mg/g shells and caffeine content of 1.76 mg/g shells, respectively.

When comparing the results with those of other studies, it should be highlighted that the composition of CBS is largely dependent on extraction conditions, such as time and temperature [26], solvent type [31], particle size, and extraction method [4], as well as the origin of cocoa [30] and agri-food practices.

## 4. Conclusions

A simple and reliable method was developed to extract theobromine and caffeine in cocoa bean shells using multivariate analysis. The mixture design allowed for optimization of extractor solution, while Doehlert design allowed for effective optimization of EGDE conditions. The optimized extraction conditions consisted of an extraction temperature of 148.5 °C and 382 s. The method presented low LODs and LOQs. The correlation coefficient, repeatability, intermediate precision, and accuracy values were in good agreement with the requirements of the developed method. In addition, the extraction procedure can be considered green, according to the AGREEprep metric tool. Therefore, EDGE proved to be very efficient in the recovery of methylxanthines from cocoa bean shells, allowing for a fast and green method of extraction.

## Figures and Tables

**Table 1:** Mixture design (simplex-centroid).

#	Ethanol (%)	Water (%)	Methanol (%)	Methylxanthines (mg g^−1^ DM)
1	50	50	0	25.3
2	50	0	50	23.7
3	0	0	100	24.5
4	0	100	0	22.7
5	100	0	0	20.6
6	33.33	33.33	33.33	25.1
7	0	50	50	23.6

**Table 2:** Coded values, real values, and respective response to the Doehlert matrix.

#	Temperature (°C)	Time (s)	Methylxanthines (mg g^−1^ DM)
1 (C)	0 (150)	0 (388)	25.9
2 (C)	0 (150)	0 (388)	28.9
3 (C)	0 (150)	0 (388)	25.8
4	1 (180)	0 (388)	17.4
5	0.5 (165)	0.86 (491.92)	17.4
6	−1 (120)	0 (388)	19.3
7	−0.5 (135)	−0.86 (284.08)	19.4
8	0.5 (165)	−0.86 (284.08)	17.8
9	−0.5 (135)	0.86 (491.92)	17.2

**Table 3:** Theobromine, caffeine, TPC, and antioxidant activities (ABTS and DPPH) for different extraction cycles under optimal conditions.

Cycle	Theobromine (mg g^−1^ DM)	Caffeine (mg g^−1^ DM)	TPC (mg GAE g^−1^ DM)	ABTS (mg TE g^−1^ DM)	DPPH (mg TE g^−1^ DM)
1	20.14 ± 0.21 ^a^	3.53 ± 0.04 ^a^	80.10 ± 2.89 ^a^	227.73 ± 4.23 ^a^	104.45 ± 1.99 ^a^
2	1.78 ± 0.02 ^b^	0.26 ± 0.01 ^b^	10.63 ± 0.53 ^b^	9.84 ± 0.95 ^b^	6.61 ± 0.54 ^b^
3	0.29 ± 0.01 ^c^	0.04 ± 0.002 ^c^	1.88 ± 0.04 ^c^	6.77 ± 0.03 ^b^	4.74 ± 0.56 ^c^

Means followed by different letters in the same column differ significantly from each other according to Tukey’s test at 95% confidence (*p* < 0.05).

**Table 4:** Validation parameters of the developed HPLC/DAD method.

Compound	Regression Equation ^a^	R^2 b^	LOD (mg L^−1^) ^c^	LOQ (mg L^−1^) ^d^	Precision ^e^		Recovery ^f^ (%) (*n* = 3)
					Intraday (*n* = 7)	Interday (*n* = 7)	
Theobromine	y = 18,972x + 31,443	0.9991	1.3	3.9	0.55	0.23	105
Caffeine	y = 29,406x − 4342	0.9996	1.1	3.4	0.65	0.67	102

^a^. In the regression equation y = ax + b, y refers to the peak area and x refers to the concentration of the analyte in mg L^−1^. ^b^. R^2^: square value of the correlation coefficient of the equation. ^c^. Limit of detection. ^d^. Limit of quantification. ^e^. Average relative standard deviation for two different measurements (10 and 30 mg L^−1^), expressed as %. ^f^. Average recoveries at two spike levels (10 and 30 mg L^−1^).

## Data Availability

The original contributions presented in the study are included in the article; further inquiries can be directed to the corresponding author.

## Abbreviations

The following abbreviations are used in this manuscript:

ANOVA	Analysis of variance
BB	Box–Behnken
CBS	Cocoa bean shell
CCD	Central Composite Design
DD	Doehlert Design
DM	Dry matter
EDGE	Energized Dispersive Guided Extraction
LOD	Limit of detection
LOQ	Limit of quantification
MAE	Microwave-assisted extraction
MD	Mixture design
TE	Trolox Equivalent
TPC	Total phenolic content
